# A proof-of-principle for decontamination of transplantation kidney through UV-C exposition of the perfusate solution

**DOI:** 10.1038/s41598-024-55574-9

**Published:** 2024-03-08

**Authors:** L. C. Goenaga-Mafud, J. D. Vollet-Filho, C. Costa, N. M. Inada, A. S. Netto, C. Kurachi, V. S. Bagnato

**Affiliations:** 1 São Carlos Institute of Physics, University of São Paulo, São Carlos, SP Brazil; 2 Department of Animal Science, College of Animal Science and Food Engineering, University of São Paulo, Pirassununga, SP Brazil; 3 Biomedical Engineering, Texas A&M University College of Engineering, College Station, TX USA

**Keywords:** Optics and photonics, Microbiology

## Abstract

Kidney transplantation is a common yet highly demanding medical procedure worldwide, enhancing the quality of life for patients with chronic kidney disease. Despite its prevalence, the procedure faces a shortage of available organs, partly due to contamination by microorganisms, leading to significant organ disposal. This study proposes utilizing photonic techniques associated with organ support machines to prevent patient contamination during kidney transplantation. We implemented a decontamination system using ultraviolet-C (UV-C) irradiation on the preservation solution circulating through pigs' kidneys between harvest and implant. UV-C irradiation, alone or combined with ultrasound (US) and Ps80 detergent during ex-vivo swine organ perfusion in a Lifeport® Kidney Transporter machine, aimed to reduce microbiological load in both fluid and organ. Results show rapid fluid decontamination compared to microorganism release from the organ, with notable retention. By including Ps80 detergent at 0.5% during UV-C irradiation 3 log_10_ (CFU mL^−1^) of *Staphylococcus aureus* bacteria previously retained in the organ were successfully removed, indicating the technique's feasibility and effectiveness.

## Introduction

Organ transplantation is among the most promising techniques for increasing the longevity and quality of life of human beings. In fact, for chronic kidney disease, transplantation is often the best treatment, in terms of improving both quality and life expectancy^1^.

According to the World Kidney Day organization, it is currently estimated that 850 million people are affected by chronic kidney disease (CKD), leading to at least 2.4 million deaths annually. In Brazil alone, more than 10 million people are living with CKD and, globally, over 2 million people undergo dialysis or live with a kidney transplant^2^. Nevertheless, the scarcity of available organs, along with organ contamination by pathogens often resistant to antibiotics, precludes their suitabilityfor donation. This situation can result in a rise in the number of patients on the waiting list and an increase in mortality rates^3^.

Consequently, the pursuit for novel methods of decontamination and verification of a renal decontamination technique motivates this study. Recently, the use of UV-C light in a circulating perfusion solution used in ex vivo lung perfusion (EVLP, a technique to preserve human lungs for transplantation) was reported^4^. Given the established safety and global application of a similar perfusion technique for kidneys^5–9^, exploring the adaptation of the UV-C irradiation to the circulatory system in kidney transplantation becomes a viable alternative.

Ultrasound (US) is another highly effective technique which, akin to UV-C irradiation, has been widely explored in different fields such as medicine, food, and industry, for its contribution in eliminating microorganisms^9^.

In fact, US applications have dual effect on microorganisms. Widely employed for microbial activation in dairy industry, US can also serve for inactivation of microorganisms depending upon factors suh as US power and frequency, sonication time, microorganism type, pH, temperature, and the presence of additional substances^10,11^. This process is achieved through the cavitation effect, involving the collapse or implosion of bubbles. This results in the production of sufficient energies capable of disrupting proteins, cell membranes, and localized heating, thereby mechanically weakening bacterial reproduction processes^10–12^.

As the detachment of microorganisms from biological substrates is not always easily achieved, the exploration of surfactants, such as Ps80 (polysorbate 80, Tween 80) becomes a viable possibility. Ps80 is a mixture of polyoxyethylene ethers of mixed oleic acid partial esters of sorbitol anhydrides^13^. Widely used as an emulsifier in both industry and laboratories, Ps80 has been documented in the literature of its inhibitory effect on plant growth and biofilm growth in various bacterial strains^13^. Additionally, it aids in the de-bonding of microorganisms^14–16^. Therefore, incorporating this nonionic surfactant in a decontamination process that relies on a circulating solution holds significant interest.

The literature also reports synergistic effects of US, UV-C, and Ps80 with other compounds in liquid media, demonstrating notable success in enhancing decontamination effects and reducing the processing time of treatments^17,18^.

In the present study, kidney decontamination for transplantation was aimed to be established. This approach has been already tested in previous studies^19^. The method involves an in vitro approach, conducting experiments for decontamination of Custodiol®, a preservation solution used in organ transplantation. The solution circulates with 5–6 log_10_ (CFU mL^−1^) of *Staphylococcus aureus* (*S. aureus*), concurrently with UV-C light irradiation, the adding of Ps80, and/or an US system. These experiments were conducted to evaluate the efficiency of decontamination and the integrity of the perfusate liquid post-procedure. Based on those results, a model of ex vivo renal contamination and decontamination of pig kidneys perfused with a LifePort® Kidney Transporter (LKT, Organ Recovery Systems, Itasca, IL) perfusion machine was proposed.

The ultimate goal of this study is to eliminate or reduce both the bacterial load of the fluid perfused through the organ and the bacterial residues that remain in the organ tissue, ensuring successful decontamination and rendering organs safe for transplantation procedures.

## Results

### *S. aureus * decontamination in PBS and HTK

**Figure 1:**
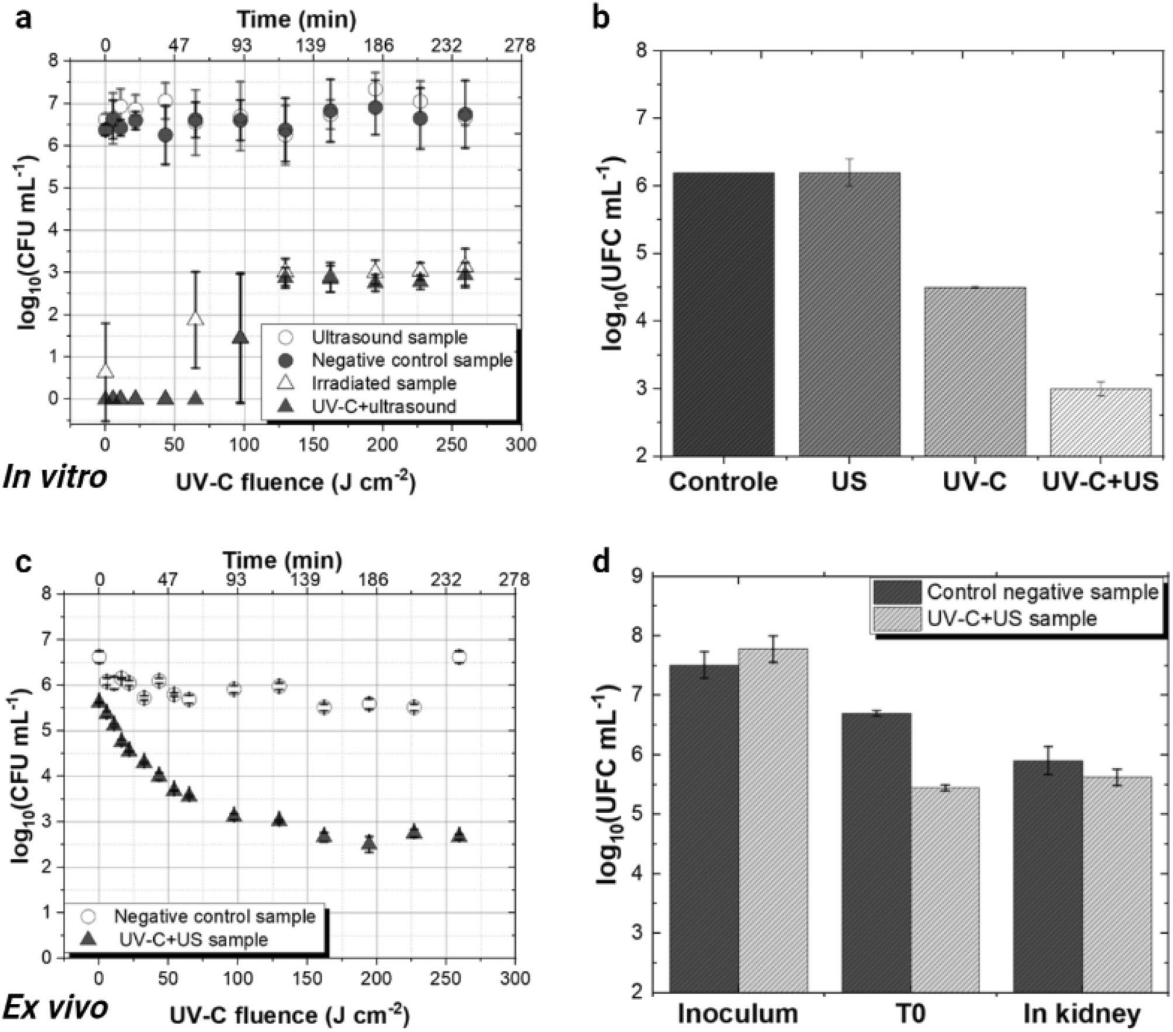
(**a**, **c)** Effect of UV-C radiation and US on the viability of bacteria (*S. aureus*) during 4 h of circulation through the decontamination system, both in vitro and ex vivo. The axes represent UV-C fluence delivered in J cm^−2^ and time in minutes, with results significantly differing from the control. Negative control samples (untreated) are shown as solid black circles, irradiated samples as open black triangles, ultrasonic samples as open black circles and the combination of the two techniques (UV-C + US) as solid black triangles. The experiment was performed in triplicate on three different occasions (n = 9), and mean values with standard deviation are presented. **(b**, **d)** Survival of *S. aureus* in the sponge and in the kidney after US bath is depicted. The bars represent control samples (untreated), US samples, samples irradiated by UV-C, and samples for the combination of the two techniques (UV-C + US). The experiment was performed in triplicate on three different occasions (n = 9).

The growth of *S. aureus *in vitro was analyzed at the conclusion of the experiment by counting the CFU mL^−1^ on Brain Heart Infusion Agar (BHI), obtained during up to 4 h of treatment as shown in Fig. 1a. In the control group, the CFU count remained stable throughout the perfusion time. Conversely, in the samples treated with the different techniques, the bacterial growth experienced rapidly reduction, typically within the first hour of liquid circulation, under light fluences up to 100 J cm^−2^. It is important to note that the entire liquid volume was not irradiated; only a section of the tube inside the decontamination system undergoes irradiation. Subsequently, a stability point was reached between the microorganism removal and decontamination rates for the same experiment, resulting in an average *S. aureus* survival fraction of 3 log_10_ (CFU mL^−1^).

The results presented in Fig. 1b describe the bacteria survival fraction following an ultrasonic bath in the renal model, comparing groups without treatment (control), US alone, UV-C irradiation alone, and the combination of both techniques. The recovery of 3 log_10_ (CFU mL^−1^) of bacteria in the combination group compared to about 4.5 or 6 log_10_ for the other groups means that the combination of the two techniques is more efficient in reducing the bacterial presence within the organ tissues. This represents an approximate 1.5 log_10_ (CFU mL^−1^) difference when compared to the UV-C results.

For the combined techniques such as US + UV-C for ex vivo decontamination in pig kidney, a 99.9% (3 log) bacterial reduction in the circulating fluid was observed (Fig. 1c). Another noteworthy outcome is related to the amount of bacteria remaining in the organ after the kidney maceration (Fig. 1d). The recovery of microbial load of negative controls was 5.9 log_10_ (CFU mL^−1^) and 5.6 log_10_ (CFU mL^−1^), corresponding to the UV-C + US groups.

**Figure 2:**
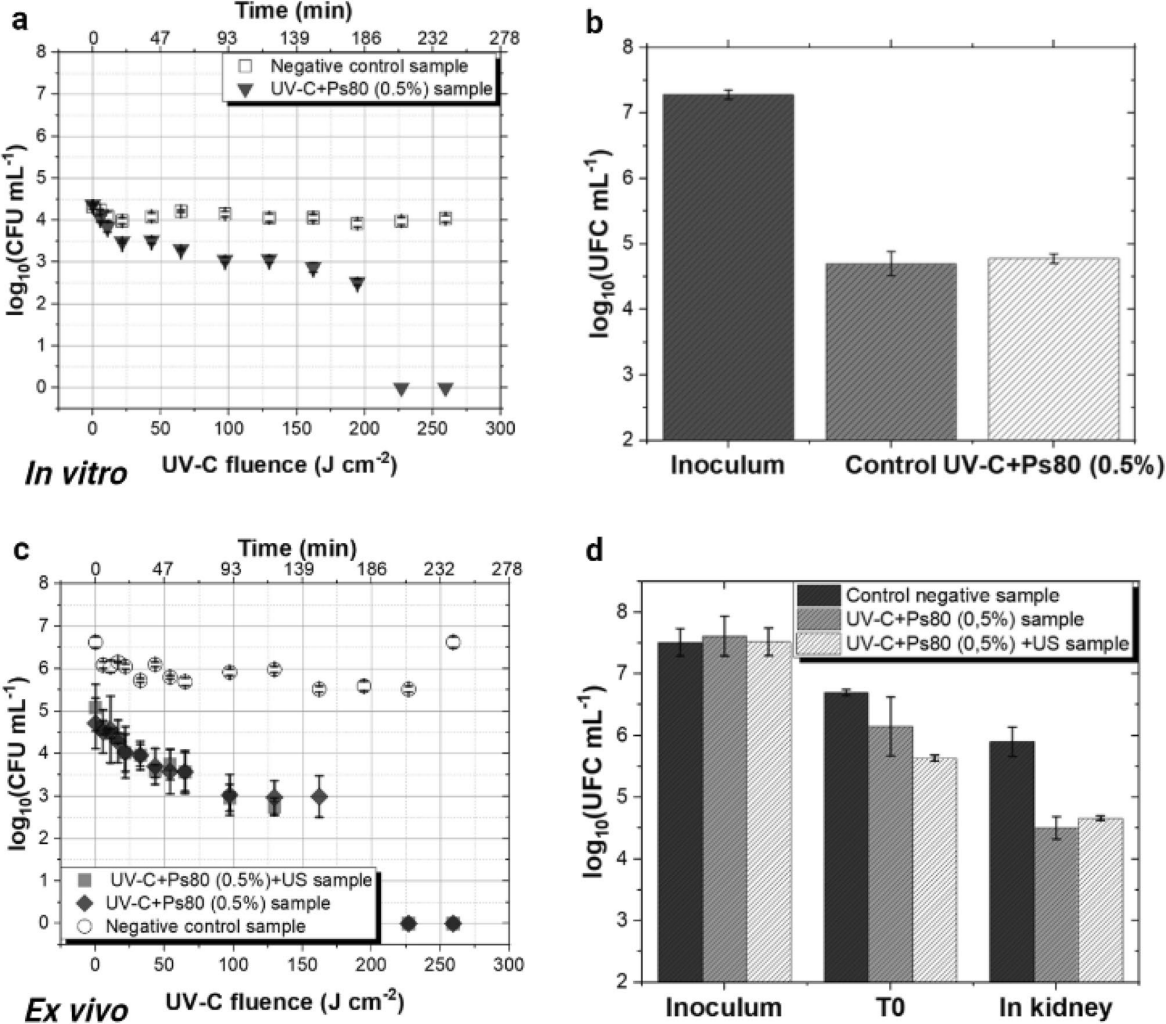
(**a**, **c**) Effect of UV-C radiation, Ps80 and US on the viability of bacteria (*S. aureus*) during 4 h of circulation through the decontamination system. The axes represent UV-C fluence delivered in J cm^−2^ and time in minutes, with results significantly differing from the control. Negative control samples (untreated) are represented as open black circles, UV-C and US combination samples as solid black triangles, UV-C and Ps80 combination samples as solid black squares, and the combination of all techniques (UV-C + Ps80 + US) as solid black diamonds. The experiment was performed in triplicate on three different occasions (n = 9), and mean values with standard deviation are presented. (**b**, **d**) Bacteria recovered from infection with *S. aureus* in the liquid. Initial *S. aureus* inoculum, T0, represents HTK with *S. aureus* bacteria after 1 h of infusion, and the macerate of kidney samples after decontamination. The experiment performed in triplicate on three different occasions (n = 9).

The introduction of Ps80 into the protocols served as an alternative approach to address the observation that the UV-C + US group has not shown the same level of efficiency ex vivo as it did in vitro. Thus, the same methodology and parameters employed in the in vitro experiments were replicated, with the addition of Ps80. The results for the groups UV-C + Ps80 in vitro and ex vivo are shown in Fig. 2. In Fig. 2a the in vitro results of the negative control group reveal consistent bacterial growth in the liquid of approximately 4 log_10_ (CFU mL^−1^) throughout the circulation process. Conversely, in the UV-C group combined with Ps80, bacterial recovery in the liquid that started at 4.5 log_10_ (CFU mL^−1^), gradually decreasing over time and UV-C exposure, until no further bacterial recover in the liquid was observed after 3 h of circulation. On the other hand, the results presented in Fig. 2b demonstrated that the combination of Ps80 + UV-C following the ultrasonic bath failed in reducing the bacterial load in the kidney model with the combination of Ps80 + UV-C.

To validate the results of Ps80 + UV-C in vitro with ex vivo experiments, in the results shown in Fig. 2c show that the control group follows a consistent pattern of recovery in the liquid throughout the decontamination process, as observed for US + UV-C experiments. The groups tested, UV-C + Ps80 and UV-C + Ps80 + US, initiated the experiment with a high microbial load in the liquid, approximately 5 log_10_ (CFU mL^−1^), which decreased over time and UV-C exposure. Notably, in the last hour of the experiment, no *S. aureus* was recovered in the liquid. In Fig. 2D, a 1.5 log_10_ (CFU mL^−1^) reduction in the microbial load of the kidney compared to the control (T0) was observed with the combination of Ps80 + UV-C. This result indicates that the UV-C + Ps80 group yields superior results compared to all tested groups, suggesting that Ps80 likely contributes to the no-adherence of bacteria within the organ tissues.

### Histological and cytotoxicity analysis

For histological analyses, slides of the untreated kidneys (control) and the kidneys treated with UV-C + Ps80 exhibited similarities in the tissues (Supplemental Fig. S1). Histopathological examination of the renal sections from the treated groups revealed no significant toxicity when compared to the control groups, indicating that the employed techniques induced no adverse effects. To assess Ps80 endothelial cytotoxicity in vitro, the MTT metabolic assay was performed 24 h after Ps80 incubation. Results showed that Ps80 at a concentration of 0.5% (v/v) led to a metabolic activity reduction of 72.4%, with statistical significance of *p* ≤ 0.001, which was comparable to the control group (Supplemental Fig. S2a). Additionally, the impact of Ps80 on morphology was evaluated via phase-contrast microscopy 24 h after Ps80 incubation (Supplemental Fig. S2b). Microscopy images revealed clear signs of cell detachment for cells subjected to Ps80 0.5% incubation, which is an indicative of cellular stress reduced metabolic activity in cells, as corroborated by MTT assay.

## Discussion

This study seeks to propose enhancements in the decontamination techniques of circulating perfusate for kidney transplantation with UV-C, incorporating additional techniques such as US application and the addition of Ps80. This research is part of a comprehensive series aimed at advancing the understanding of the key aspects of this technique, with potential implications for future clinical approaches.

All contamination experiments were conducted with *S. aureus*, a bacterium significant clinical importance due to its diverse nosocomial disease manifestations and its propensity to develop antibiotics resistance^20,21^. Furthermore, *S. aureus* can form biofilms within the kidney, which makes it a pertinent target for decontamination efforts^22^.

UV-C decontamination is a highly successful technique extensively applied in medicine and food industry^23,24^. Its implementation in organ decontamination is a novel and evolving area of exploration. Recent studies of kidney decontamination for transplantation using UV-C^19^, revealed limitations, demonstrating its inability to completely eradicate bacteria from the kidney during the perfusion process. In order to address and overcome such limitations, in the present study additional physical and chemical techniques were incorporated, namely US and Ps80 detergent, respectively.

US is a technique that can provide powerful disinfection by itself. However, its suitability for large-scale microbiological decontamination requires further evaluation. When employed in conjunction with other technologies, it is expected to yield excellent results^25^. Hence, this study explores the potential of combining US with UV-C for enhanced efficacy.

The inactivation of microorganisms occurs at low frequencies and high sonoporation degrees. Sonoporation induces the formation of pores in the membrane, destabilizing porins and lipopolysaccharide molecules in the cell membrane, ultimately leading to microbial death through the leakage of cell contents and membrane rupture or alternation^10^. Conversely, lower degrees of sonoporation may enhance microbial growth by facilitating the removal of cellular residues and promoting the transfer of oxygen and nutrients necessary for cell growth^26–28^. This may be occurring in the ex vivo results, as the combination of techniques was not successful, showing increased adhesion of the bacteria to the tissues. Also, it must be noted that the organ was not in direct contact with US, which hinders the mechanism of action of US in preventing the bacterial adherence. Moreover, *S. aureus* being a bacterium with high cellular adhesion, it occasionally functions as an intracellular pathogen, exhibiting the ability to adhere to cellular surfaces and undergo internalization^29^.

On the other hand, surfactants are amphiphilic molecules, as they contain both hydrophilic and hydrophobic portions that can reduce surface tensions and primarily accumulate mainly at interfaces, such as cell membranes. They have demosntrated the ability to inhibit the adhesion of various microorganisms^30,31^. The cell wall or membrane serves as an interface through which microorganisms interact with their surroundings. These interfaces play a pivotal role in controlling the mass transfer of nutrients, wastes and signaling molecules. Furthermore, they also influence microbial growth rates by sequestering toxic metabolites, buffering pH, or by reducing substrate availability^31^.

Several studies exploring the effects of Ps80 at low concentrations aim to modify the physical properties of the surface for *S. aureus* and other microorganisms, making it anti-adhesive. Others focus on creating a surface with inherent antibacterial properties^16^.

Studies report surfactants can exert toxic effects on microorganisms by inducing membrane disruption, leading to cell lysis. This disruption can increase membrane permeability, causing the leakage of metabolites. Surfactants can also alter the physical structure of the membrane or disrupt protein conformations, thereby interfering with critical membrane functions such as energy generation and transport^31^.

Historically, various studies indicated that chemical surfactants toxicity and/or efficacy as antimicrobial agents depend on their charge (cationic, anionic and non-anionic)^32,33^. Glover et al., conversely, examined the effects of four chemical surfactants’ impact on various microbes, including *S. aureus*, and found that increased membrane fluidity resulting from surfactant exposure did not consistently correlate with toxicity^34^.

Histological analysis revealed similar outcomes in terms of inflammatory infiltrates, reduced glomerular space between glomerular tuft and Bowman's capsule, and mild tubular swelling in both the control and Ps80% + UV-C groups. The observed similarities between the control and treated samples make it challenging to attribute the identified changes solely to the effects of Ps80 on the organ.

A study focusing on post-mortem inspection of pig kidneys intended for trade and human consumption was conducted in slaughterhouses, and revealed kidneys exhibiting lesions such as areas of infarction, congestion, chronic interstitial nephritis, tubular epithelial swelling, and multifocal lymphohistiocytic infiltrate. These lesions are relatively common in swine species and seem to lack clinical relevance^35–37^. The kidney is an organ susceptible to hypoperfusion, which may induce tubular cell death, water fasting, and solids before slaughter, which are also consequences of necrosis in the studied tissues^38^.

Concerning Ps80 cytotoxicity, although the MTT assay indicated a 72.4% reduction in metabolic activity of endothelial cells compared to the control group, the observed cytotoxic effects in vitro did not manifest in the ex vivo histopathology analysis of treated kidney tissue. In other words, there was no evidence of cytoplasmic degeneration or necrotic foci in the kidney tissue. This suggests that endothelial cells in an in vitro environment are more vulnerable to the cytotoxic effects of Ps80 compared to the cells comprising the complex structure of the kidney tissue.

Studies conducted in mice to evaluate the impact of Ps80-coated nanoparticles as a potential therapy against breast cancer showed no cytotoxicity in cells and no histological changes associated with toxicity in various vital organs, including the kidneys^16^.

The results of this study align with the more recent findings in the literature, suggesting that non-ionic surfactants, such as Ps80, may represent important agents for enhancing antimicrobial efficacy action when combined with UV-C, which may be linked to a potential compromise in membrane function.

It is noteworthy that this decontamination process serves as an adjunct technique to the current perfusion process carried out using hypothermic machine perfusion devices. Essentially, our approach introduces only a quartz-windowed bypass, a pump, and an ultraviolet-C light source for the decontamination circulation. Given that ultrasound has not demonstrated superior improvement on reduction compared to the addition of Ps80, the incorporation of Ps80 is the actual modification to the mentioned approach. Integrating this pump-bypass-light source assemble into the kidney preservation device along with Ps80 is estimated to increase the overall system cost by no more than 10%, thus impacting the preservation process minimally.

## Materials and methods

### Bacterial preparation

For the experiments, a *S. aureus* ATCC25923 strain was used, incubated in BHI broth for 18 h with agitation at 150 rpm and a temperature of 37 °C^19^.

### Decontamination system

**Figure 3:**
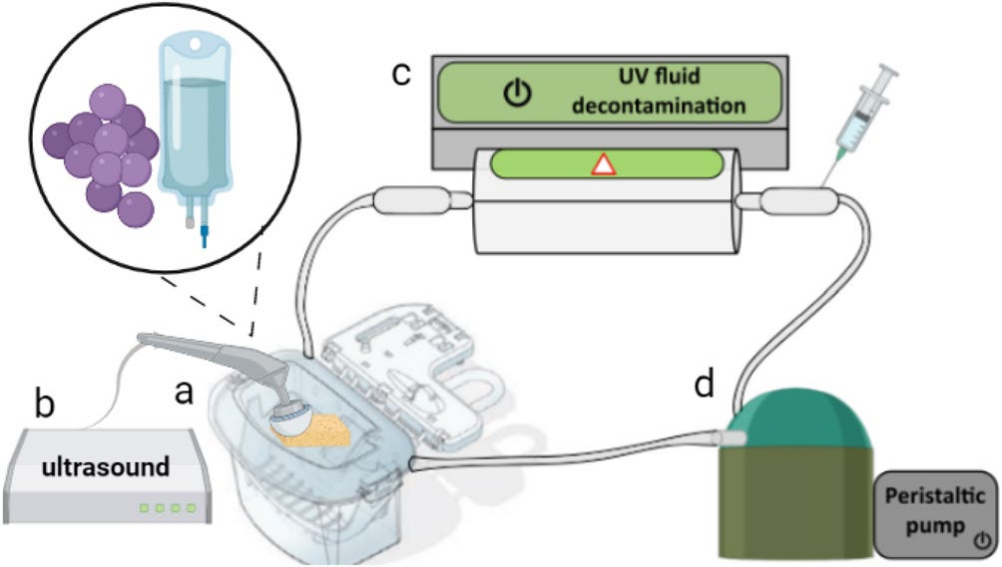
Scheme for UV-C decontamination system. The solution was maintained in a reservoir (**a**) and pumped through the circulation circuit. In the reservoir, the solution undergoes decontamination by the US (**b**) and was then pumped into the tube that passes through the UV-C light source (**c**). Subsequently, the solution passed through the peristaltic pump (**d**) and was delivered back to the reservoir.

The prototype of the UV-C light decontamination system was developed by the Technological Support Laboratory (LAT, São Carlos Institute of Physics—University of São Paulo) and comprises UV-C lamps emitting at 254 nm. The circulation circuit consists of silicon tubes and a peristaltic pump (WP10, Welco, Japan) with a flow rate circulation capacity of 200 mL min^−1^ (For additional details, refer to Goenaga-Mafud, L. C, 2022)^19^. The system also incorporates ultrasound (Sonopulse III, IBRAMED, Amparo/SP, Brazil) with a total intensity of 2.0 W cm^−2^ and frequency of 1 MHz. A schematic of the decontamination system is illustrated in Fig. 3.

### In vitro kidney retention model decontamination in HTK

The renal model was employed to simulate, to some extent, the resistance created by the kidney during perfusion^19^. A commercial cleaning multi-purpose sponge (Scotch-Brite®, 3 M do Brasil Ltda, Sumaré/SP, Brazil) measuring 110 mm × 75 mm × 20 mm was utilized. The sponge was directly contaminated by *S. aureus* and immersed in one liter of HTK solution, deposited in a single-use kit, customized to be used as a reservoir for containing the kidney in the Lifeport® Kidney Transporter perfusion machine. The US system was adapted to the cassette for sonification. The cassette was also connected to the decontamination system (Fig. 3) via the silicone tubes to enable UV-C irradiation. The solution was circulated under four regimens: with the UV-C lights on (“UV-C irradiated” group), US on (“ultrasound irradiated” group), a combination of the two techniques on (“UV-C + ultrasound” group), and off (“negative control” group) for 4 h. Solution samples were collected at times 0, 5, 10, 20, 40, 60, 90, 120, 150, 180, 210, and 240 min and then diluted for bacterial quantification (colony forming units per mL, CFU mL^−1^) using the dispersion plate method. After circulation, to quantify the bacteria remaining on the sponge, an ultrasonic bath was performed on the sponge. The collected samples were diluted and the bacteria were quantified using the dispersion plate method. Each experiment in this investigation was performed in triplicate and repeated on three separate occasions (n = 9).

### Ex vivo kidney perfusion and decontamination

To assess how an actual organ would respond to decontamination and how the results would align with the in vitro experiments conducted using the decontamination model for the circulating preservation solution^19^, a single ex vivo swine (weighing approximately 50 kg) kidney perfusion and decontamination was conducted for each group. A kidney, obtained from a local slaughterhouse animal, was cannulated in the renal artery and heparinized with 400 IU kg^−1^ of sodium heparin, within 30 min after its removal. Subsequently, the organ was placed in the cassette, connected to an LKT machine, and perfused with 1 L of saline solution for an initial organ flush. Concurrently, the cassette was connected to the decontamination system (Fig. 3), to expose the solution to UV-C irradiation for a 4-h perfusion.

The machine perfusion parameters were set for 30 mmHg pressure and a 60 mL min^−1^ flow rate, while maintaining temperature between 2 and 8 °C. After 1 h, the circulating saline solution was replaced with 1 L of the contaminated HTK solution (5–6 log_10_ units in CFU mL^−1^). This step was undertaken to intentionally contaminate the kidney, allowing for subsequent decontamination of the microbial load residing within the kidney itself. The machine operated in “infusion mode” for an additional 1 h to ensure kidney contamination.

It is essential mentioning that no ethics committee is required for those experiments, as they involve the use of a discarded organ and do not entail the use of the entire animal for experimentation.

### Decontamination system with US in the perfusion liquid

The decontamination and US systems were connected to the machine by the cassette, without interfering with the kidney perfusion pumping promoted by the LKT machine. The US parameters employed were 1 MHz intensity, continuous frequency of 2.5 W cm^−2^, maintained on for 30 min and off for 5 min (to prevent overheating), repeating this cycle until the completion of the 4 h of organ perfusion. Samples of the circulating solution were collected at times 0, 5, 10, 20, 40, 60, 90, 120, 150, 180, 210, and 240 min, throughout the 4 h of perfusion and decontamination. Subsequently, the 300 g kidney was completely macerated, and three samples were collected. All samples were diluted with PBS and the spread-plate method was used to determine the bacterial count during irradiation, as well as the remaining microbiological load in the organ after the decontamination protocol.

### Decontamination system with Ps80 in the perfusion liquid

For ex vivo decontamination with UV-C and Ps80, the same protocol described in section “Decontamination system with US” was followed for organ decontamination, with the addition of 50 ml of Ps80 at 10% to the 1 L of contaminated HTK solution containing 8 log_10_ units in CFU mL^−1^. This addition was made to achieve an HTK concentration with Ps80 at 0.5%.

### Decontamination system with US and Ps80 in the perfusion liquid

For this group, all techniques—UV-C, US, and Ps80—have been combined, as described in the methodologies mentioned above.

### Histological analysis

Kidney tissues were collected before maceration from negative control group and Ps80 + UV-C irradiated group. Each treated kidneys group was fixed in 10% neutral buffered formalin within 30 min of resection, and after 24 h of fixation, the formalin solution was changed to 70% alcohol and sent for histological analysis (PatVet Veterinary Pathology Laboratory, São Carlos, SP, Brazil). The staining method used was hematoxylin and eosin (HE), and all fragment analyses and photomicrographs were obtained in an Opticam binocular optical microscope (model O400S), with 10 × and 40 × objectives.

### Cytotoxicity analysis

#### Cell culture

Experiments regarding to Ps80 cytotoxicity were conducted using the HUVEC EA.hy926 (ATCC CRL-2922™) cell line. This cell line, an established somatic cell hybrid of primary human umbilical vein cells and lung epithelial carcinoma (A549 ATCC CRL-185™) cells, is widely used in cardiovascular disease research due to its similarity to primary endothelial cells. The cells were cultured in Dulbecco's Modified Eagle's Medium (DMEM) supplemented with 10% (v/v) fetal bovine serum (FBS) in a humidified incubator at 5% CO_2_ and 37 °C. The culture medium was replaced three times a week, and subculture was performed whenever necessary, following the recommended protocol of rinsing with PBS 1X at pH 7.4, flask detachment with 0.25% (w/v) Trypsin – 0.53 mM EDTA, and supplementing with (FBS)-supplemented medium for trypsin inactivation.

#### Ps80 cytotoxicity—MTT assay and cell morphology

MTT Assay comprises a colorimetric mitochondrial activity assay, in which a yellow salt (MTT) is reduced to formazan purple crystals by mitochondrial reductases. Therefore, this assay serves as an indirect method to evaluate metabolic activity, i.e., cytotoxicity. In these experiments, cells were inoculated in 96-well plates (1 × 10^4^ cells, 100 µL per well) 24 h before Ps80 incubation. After this interval, the medium of each well was replaced with another 100 µL of DMEM 10% (v/v) FBS containing Ps80 at concentration of 0.5% (v/v), and maintained for additional 24 h in a humidified incubator at 5% CO_2_ and 37 ºC. The MTT assay was performed after this time, adding 100 µL of MTT at 0.5 mg/mL dissolved in DMEM 1% FBS without phenol red during 3 h. The formazan crystals were then diluted with 100 µL dimethyl sulfoxide (DMSO). The absorbance of the resulting solution was immediately recorded in a spectrophotometer (MultiskanTM FC Microplate Photometer, Thermo Fisher Scientific, USA) at 570 nm and 690 nm. Three independent experiments were conducted with N = 9 per group per replicate. Results were presented as mean ± standard deviation. Means were compared pairwise using Tukey's Test. Cell Morphology was analyzed by phase-contrast microscopy 24 h after Ps80 incubation, using the inverted microscope ZEISS Axio Observer.Z1, Carl Zeiss.

### Statistical analysis

All experiments were performed in triplicate, and standard deviations were calculated. Statistical analysis to compare the different cell viabilities within the same group and identify significant differences between techniques was conducted with Prisma (GraphPad software) with the two-way ANOVA and the Dunnett's multiple comparisons tests. In all tests, results were considered significantly different with a *P*-value < 0.0001.

## Conclusion

In conclusion, this study highlights improvements in the process of circulating decontamination of swine kidneys in HTK preservation solution through irradiation with ultraviolet light at 254 nm in combination with Ps80.

US in this study was not successful in removing microorganisms. Despite that, UV-C remains an excellent technique for decontaminating *S. aureus* in the liquid, reducing about 100% of the microbial load in the preservation solution in less than 2 h of irradiation during organ perfusion. The combination of Ps80 with UV-C improved the technique, reducing up to 1.5 log_10_ (CFU mL^−1^) of microorganisms retained in the organ after perfusion.

This was considered a satisfactory result, which may help to overcome the limitations of the technique in decontaminating the organ itself, as observed in the initial studies (19). Such an achievement shall provide improvements that accelerate the release of microorganisms trapped in the organ. The histological and cytotoxicity analyses showed that the use of the techniques does not alter the physiology of the organ after the process, a priori, but more tests should be performed with pig kidneys that are in healthy and controlled conditions. The use of the technique is promising for the decontamination process of a transplant organ by irradiating the circulating preservation solution during perfusion with UV-C and Ps80.

### Supplementary Information

Supplementary Information.

## Data Availability

The data used and/or analyzed during this study are available from the corresponding author on reasonable request.
