# Supplementary material for: A proof-of-principle for decontamination of transplantation kidney through UV-C exposition of the perfusate solution
Source: Sci Rep. 2024 Mar 8;14:5715. doi: 10.1038/s41598-024-55574-9 (PMC10923919; doi:10.1038/s41598-024-55574-9)
Supplement: Supplementary file 1 — Supplementary Information. [file 41598_2024_55574_MOESM1_ESM.docx]

**Supplementary Information**

The histological slides of the untreated kidneys (control) and the kidneys treated with UV-C + Ps80 showed similarities in the tissues, such as normal structure of the hepatocytes, no signs of cytoplasmic degeneration and necrotic foci, with most of the glomeruli healthy and abundant capsular space and tubules, as well as features of toxicity such as leukocyte infiltration, edema exudate, necrotic foci, and distorted glomeruli.

Supplemental Figure S1 shows some discrete signs of damage found in both control and treated tissues. In S1a and S1b, few inflammatory cells are observed in control and treated groups. In S1c and 1Sd, slight signs of tubular swelling are observed. In S1e and S1f, small reduction of the space between the glomerular tube and Bowman's capsule was observed in both control and treated groups samples. Similarly, histopathology of the renal sections from the treated groups revealed no significant toxicity compared to the control groups that might be caused by the techniques used in the treatment.


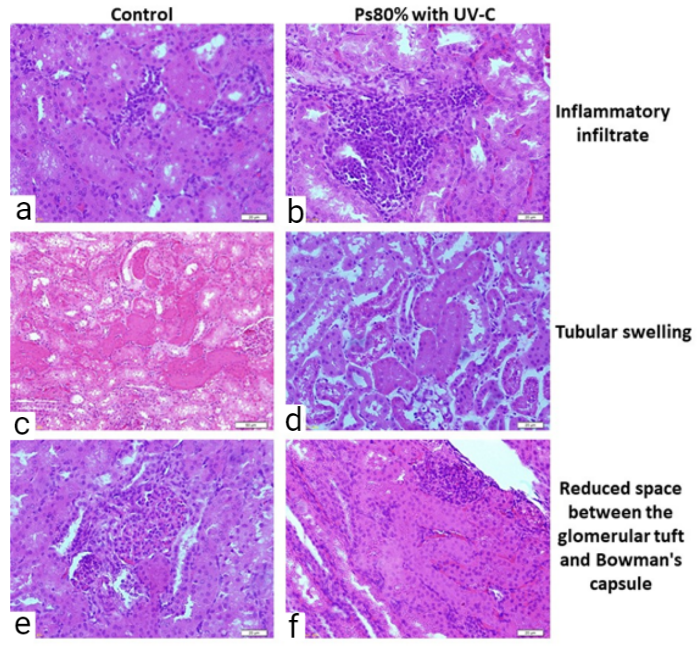


**Supplemental Figure S1.** Photomicrographs of pig kidneys (control and treated groups), with findings as shown in (A) to (F). **(a)**. presence of a focus of interstitial lymphoplasmacytic inflammatory infiltrate in mild degree, control group (40x); **(b)**. a focus of lymphoplasmocytic inflammatory infiltrate, treated group (40x). **(c)**. identification of foci of tumefaction of the lining epithelium of the collecting tubules of the cortical zone, control group (20x); **(d)**. identification of a focus of tumefaction of the lining epithelium of the collecting tubules of the cortical zone, treated group (40x). **(e)**. reduction of the space between Bowman's capsule and glomerular tuft, control group (40x); **(f)**. reduction of the space between Bowman's capsule and glomerular tuft, treated group (40x).

For Ps80 endothelial cytotoxicity in vitro, the MTT metabolic assay was performed 24 h after Ps80 incubation. It was observed that Ps80 at a concentration of 0.5% (v/v) resulted in a metabolic reduction of 72.4% with statistical significance of p≤0.001, being comparable to the control group (Supplemental Fig. S2a). Additionally, the effects of Ps80 on morphology were assessed by phase contrast microscopy 24 h after Ps80 incubation (Supplemental Fig. 2b). Microscopy images showed clear signs of cell detachment for cells submitted to Ps80 0.5% incubation, which is due to the cellular stress and the decrease in cell metabolic activity caused by this condition, as observed by MTT assay.


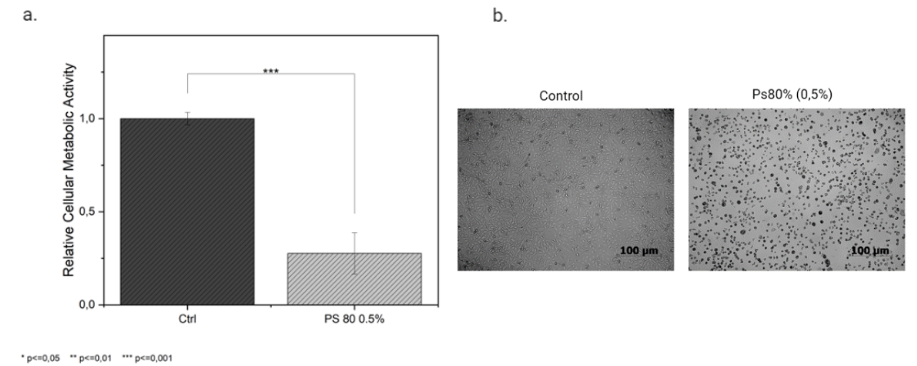


**Supplemental Figure S2. (a).** Ps80 Cytotoxity of Ps80 (0.5%) on HUVEC (EA.hy926) with the control sample. **(b)**. Cytotoxicity of Ps80 (0.5%) on HUVEC (EA.hy926) morphology by phase contrast microscopy with the control sample.
